# Combined phacoemulsification and viscocanalostomy with Ologen implant versus combined phacoemulsification and viscocanalostomy

**DOI:** 10.1186/s12886-019-1049-6

**Published:** 2019-02-06

**Authors:** Ahmed A. M. Gad, Bahaa-Eldin Hasan Abdulhalim, Ayman Lotfy, Ayman Mohamed Abdelrahman, Ahmed Samir Ahmed

**Affiliations:** 1 Ophthalmology Department, Zagazig University, Zagazig, 44511 Egypt; 2 Ophthalmology Department, Shaqra University/Zagazig University, Shaqraa, Kingdom of Saudi Arabia

**Keywords:** Viscocanalostomy, Ologen, Glaucoma, Phacoemulsification

## Abstract

**Background:**

To study the efficacy of the biodegradable collagen implant Ologen® as an adjuvant in phaco-viscocanalostomy in patients with coexisting cataract and primary open angle glaucoma.

**Methods:**

This prospective, interventional, randomized clinical study was done at Alpha Vision Center, Zagazig, Egypt. Patients with coexisting cataract and glaucoma were randomized to receive either phaco-viscocanalostomy (Phacovisco group) (39 eyes) or phaco-viscocanalostomy with Ologen® implant (OloPhacovisco group) (40 eyes). Follow-up period was 2 years. Nd:YAG laser goniopuncture was done in cases where the intraocular pressure (IOP) was elevated above 21 mmHg after discontinuation of corticosteroid eye drops at any follow-up visit.

**Results:**

No significant operative or postoperative complications (other than failure) were encountered in either group. At 2 years follow-up, the mean IOP level was statistically significantly decreased in the OloPhacovisco group (*p* = 0.02) and complete success occurred in 23 eyes (59.0%) in the Phacovisco group and in 32 eyes (80.0%) in the OloPhacovisco group. There was a statistically significant higher success rate regarding complete success in patients that received Ologen® implant (*p* = 0.04).

**Conclusions:**

Ologen® implant improved the success rate of phaco-viscocanalostomy. Larger studies with longer follow-up periods may be required to confirm these findings.

**Trial registration:**

This trial was retrospectively registered on 20/12/2018 under the number (NCT03782051).

## Background

Surgical treatment of glaucoma is indicated in cases of failure of medical treatment. Trabeculectomy is considered the standard surgical treatment of glaucoma. Non-penetrating glaucoma surgeries, e.g. viscocanalostomy and deep sclerectomy, are becoming good alternatives. Viscocanalostomy has fewer complications than trabeculectomy, e.g. bleb leak, hypotony and bleb-related infections [1–4].

Since the prevalence of both cataract and glaucoma increases with age, phakic patients who are scheduled for glaucoma surgery may also require cataract extraction, or patients scheduled for cataract surgery may be scheduled for combined surgery. Improvement of phacoemulsification techniques and stable anterior chamber depth with non-penetrating glaucoma procedures may favor combined viscocanalostomy and cataract surgery in patients with glaucoma and a coexisting cataract [4].

A biodegradable porous collagen-glycosaminoglycan copolymer matrix implant (Ologen®) is a simple chemical analog of extracellular matrices and has been used as an adjuvant to increase the long-term success of trabeculectomy. The degradation time of this type of implant is around 180 days; this implant consists of porcine based, lyophilized, crosslinked type I atelocollagen (≥ 90%) and glycosaminoglycans (≤ 10%). During trabeculectomy surgery it is placed subconjunctivally and acts as a spacer to mechanically separate the subconjunctival and episcleral tissues to decrease subconjunctival fibrosis [1, 3].

The purpose of this study was to evaluate the use of Ologen® implant as an adjuvant in phaco-viscocanalostomy in patients with coexisting cataract and glaucoma. Our hypothesis is that using Ologen® implant as a spacer in the subscleral reservoir in phaco-viscocanalostomy reduces fibrosis and increase the success rate of this operation.

## Methods

This study included 40 eyes from 40 patients that had primary open-angle glaucoma (POAG) with age-related cataract. The patients were recruited from the outpatient clinics of Alpha Vision Center, Zagazig, Egypt between August 2013 and January 2016. The study was approved by the Ethical Committee of Alpha Vision Center. The study adhered to the tenets of the Declaration of Helsinki.

Cases were included if they were ≥ 40 years old at the time of surgery, with POAG and age-related cataract. The diagnosis of open-angle glaucoma was based on history, clinical examination and typical glaucomatous visual field loss by Humphrey field analyzer (HFA; Carl Zeiss Meditec Inc., Oberkochen, Germany).

The indications for surgery were the presence of significant cataract interfering with vision (visual acuity ≤0.5) in the presence POAG. Patients were included if cataract was associated with uncontrolled glaucoma, (IOP > 21 mmHg despite maximally tolerated medical therapy) or if the IOP was ≤21 mmHg with use of at least two antiglaucoma drugs with medication intolerance, poor patient compliance, patients could not attend medical supervision or had visual field deterioration.

Patients were excluded if they had closed-angle glaucoma, other types of open angle glaucoma (OAG), e.g. pigmentary glaucoma, inflammatory glaucoma or neovascular glaucoma, previous ocular trauma or surgery, lens subluxation or other eye diseases affecting the vision, e.g. anterior uveitis. Patients were also excluded if there was a large perforation of the Descemet’s membrane with iris prolapse during surgery (cases with microperforation, which is defined as small perforation with no associated iris prolapse, occurring during surgery were not excluded) or if they had other intraoperative complications that might affect the IOP, e.g. vitreous loss.

A written informed consent was obtained from every patient after explanation of the procedure and the possible consequences of the surgery.

Eligible patients were randomized (using a computer-assisted program) to receive either phaco-viscocanalostomy (Phacovisco group), or phaco-viscocanalostomy with Ologen® implant (OloPhacovisco group).

### Patient examinations

Patient histories were taken including age, gender, previous ocular surgery, trauma or any previous ocular inflammation, e.g. keratitis, iridocyclitis, etc. Slit-lamp examination was done for examination of anterior chamber angle, determination of the type of glaucoma and the degree of cataract, measuring IOP (using Goldmann applanation tonometer), assessment of optic nerve head and retinal examination, if possible. Visual acuity (VA) was expressed in decimal fraction.

### Surgical technique

All operations were carried out by (AAMG) using peribulbar anesthesia.

After phacoemulsification, a traction suture in the cornea was made using 8–0 polyglactin 910 (Vicryl®, Ethicon Inc., Bridgewater, NJ, USA).

**Fig. 1:**
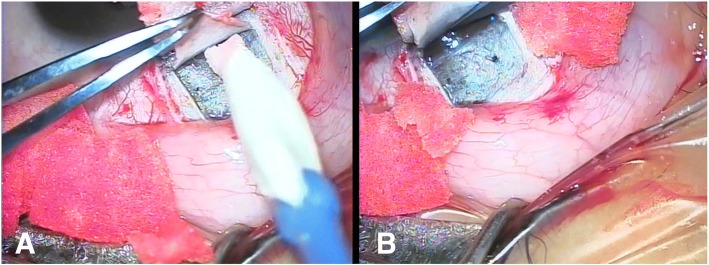
Deep flap dissection. **a** Blunt dissection of deep scleral flap with microsponge to make a Descematic window. **b** Removal of deep flap by Vannas scissors

A fornix based conjunctival flap was fashioned and no cautery was applied. Hemostasis was only achieved by using a microsponge soaked with 1/100,000 adrenaline. A superficial scleral flap of 5 × 5 mm was made and dissection was done 2 mm into the clear cornea. A smaller deeper flap of 4 × 4 mm was made until deroofing of Schlemm’s canal was achieved. Decompression of the eye was done through one of the paracentesis incisions, then blunt dissection with a microsponge was done to create a descematic window that extended over Descemet’s membrane until 1 to 2 mm inside the clear cornea. Subsequently, the sides of the deep flap were freed from the adjacent sclera by Vannas scissors and the deep flap was cut by the same scissors (Fig. 1).

The juxtacanalicular trabeculum was removed and then the two openings of Schlemm’s canal were cannulated on both sides using a viscocanalostomy cannula (Eagle labs, Rancho Cucamonga, CA, USA) and high-viscosity sodium hyaluronate (Healon GV; Abbott Medical Optic Inc., Santa Ana, CA, USA) was injected into the canal on both sides.

In the Phacovisco group, the superficial flap was closely sutured using four interrupted 10–0 nylon sutures, and then Healon GV was injected into the subscleral lake.

**Fig. 2:**
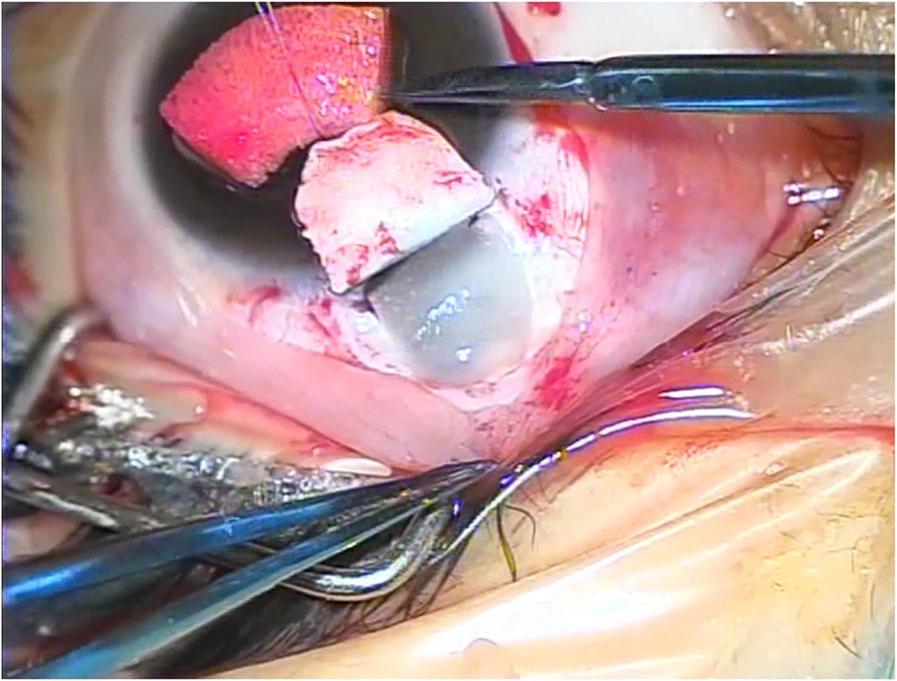
Ologen insertion. Insertion of the biodegradable Ologen® implant at the site of the removed deep scleral flap

In the OloPhacovisco group, a 4 × 4 mm piece of Ologen® (Model: 862051 “12mm(D) x 1mm (H)”) (Aeon Astron Europe B.V., Leiden, The Netherlands) was fashioned to be nearly of the same size as the cut deep scleral flap to fit inside the subscleral reservoir (Fig. 2). Then the superficial flap was sutured tightly over it using four interrupted 10–0 nylon sutures (Ethilon®, Ethicon Inc., Bridgewater, NJ, USA). Some air was injected from the paracentesis inside the eye. Then conjunctiva was closed using 8–0 polyglactin 910 sutures (Vicryl®, Ethicon Inc., Bridgewater, NJ, USA).

Postoperative management included topical administration of 0.5% moxifloxacin drops with a tapered schedule of 1% prednisolone acetate. Antiglaucoma medication was ceased after surgery in all cases.

Postoperatively, follow-up examination was done on days 1, 7, 14 and 30, then every 2 months for 2 years. The follow-up examination included assessment of IOP, VA and slit-lamp examination. Postoperative complications were recorded. Signs of inflammation, such as cell infiltration and flare, were recorded and graded from 0 to 4.

Nd:YAG laser goniopuncture was considered in all cases with elevated postoperative IOP above 21 mmHg after discontinuation of corticosteroid eye drops for at least 14 days. The Nd:YAG goniopuncture was performed using an Ellex Super Q YAG laser machine (Ellex Medical Lasers Ltd., Mawson Lakes, SA, Australia) and the gonioscopy mirror of Goldman three mirror universal laser lens (Ocular, Bellevue, Washington, USA) with a laser power setting of about 3 to 6 mJ and using about 2 to 15 shots aiming at the opening of the trabeculo-Descement window, the number of laser shots needed to open the Descemet’s membrane was counted and recorded in each group. Antiglaucoma drugs were prescribed if the IOP was elevated above 21 mmHg after considering Nd:YAG laser goniopuncture.

The main outcome measures were the IOP at the 2-year follow-up and the overall success, which included complete success when IOP was ≤21 mmHg without any antiglaucoma drugs, and qualified success if IOP was ≤21 mmHg with the use of a single antiglaucoma medication. Failure was considered if IOP ≤ 21 mmHg could not be reached even with the use of a single antiglaucoma medication and after performing Nd:YAG laser goniopuncture, at any follow-up visit. The secondary outcome measures were surgical complications, the use and results of Nd:YAG laser goniopuncture and visual acuity results. The last data for failed cases were used for further statistical analysis.

### Statistical analysis

The data were analyzed was using the software Statistical Package for Social Science (SPSS) version 16.0 (SPSS Inc., Chicago, Illinois, USA). Continuous variables were expressed as mean ± SD and compared using student t tests; two tailed test was used to detect significance between the two groups and one tailed t test was used to detect the significance before and after intervention in the same group. Mann-Whitney U test was used for nonparametric analysis. Categorical variables were expressed as percentages and were analyzed using the chi square (χ^2^) test. A value < 0.05 was considered statistically significant.

## Results

Forty-one eyes were allocated to the Phacovisco group and 40 eyes were allocated to the OloPhacovisco group. Two eyes in the Phacovisco group had perforation of Descemet’s membrane with iris prolapse during surgery and were excluded. No cases were complicated with vitreous loss. The remaining 39 cases in the Phacovisco group and 40 cases in the OloPhacovisco group completed the follow-up period. There were no significant differences between the two groups regarding patient demographics or preoperative data (Table 1).

**Table 1:** Demographic and preoperative data, and follow-up period of the studied groups

Parameters	Phaco-viscocanalostomy group (*n* = 39)	Phaco-viscocanalostomy with Ologen® implant group (*n* = 40)	*p* value
Age; yrs			
Range	40–81	41–79	
Mean ± SD	57.95 ± 9.61	58.18 ± 9.00	0.91^†^
Sex; n (%)			
Male	16 (41.0)	17 (42.5)	
Female	23 (59.0)	23 (57.5)	0.89^‡^
Visual acuity			
Range	0.1–0.7	0.1–0.6	
mean ± SD	0.41 ± 0.18	0.37 ± 0.17	0.33^†^
IOP^a^; mmHg			
Range	15–25	15–26	
mean ± SD	19.46 ± 3.26	19.18 ± 3.19	0.69^†^
Antiglaucoma drugs; n			
Range	2–4	2–4	
mean ± SD	2.85 ± 0.87	2.88 ± 0.91	0.70^§^

*IOP* intraocular pressure, *SD* standard deviation

^a^measured with treatment

^†^Two-tailed student t test

^‡^Chi square test

^§^Man-Whitney U test

No significant operative complications (other than the two case of anterior chamber perforation) were encountered in either group. The average operative time was 35 min, which was slightly longer in the OloPhacovisco group. No postoperative complications were encountered during the 2-year follow-up.

During the follow-up period, number of cases in both groups had IOP > 21 mmHg after discontinuation of corticosteroid eye drops, for whom Nd:YAG laser goniopuncture was indicated; they were 21 cases (53.8%) in the Phacovisco group and 22 cases (55.0%) in the OloPhacovisco group. There was no statistically significant difference in their pre-laser goniopuncture IOP (*p* = 0.76). The mean IOP after goniopuncture was statistically significantly lower (*p* = 0.03), and the mean reduction of IOP after goniopuncture was also significantly lower (*p* = 0.03) in the OloPhacovisco group in comparison to the Phacovisco group. The Nd:YAG goniopuncture succeeded to make a statistically significant reduction in IOP to ≤21 mmHg in 5 of 21 cases in the Phacovisco group and 13 of 22 cases in the OloPhacovisco group (*p* <  0.02 in each group). Comparing the number of laser shots needed to first open the Descemet’s membrane in the two groups, we found that in the OloPhacovisco group the mean number of laser shots was statistically significantly lower than in the Phacovisco group (Table 2).

**Table 2:** Intraocular pressure (mmHg) results in cases with goniopuncture

Parameters	Phaco-viscocanalostomy group (*n* = 21)	Phaco-viscocanalostomy with Ologen® implant group (*n* = 22)	*p* value^*^
IOP pre-goniopuncture			
Range	22–45	22–44	
mean ± SD	28.10 ± 6.39	28.73 ± 6.29	0.76
Number of shots			
Range	8–16	2–9	
mean ± SD	11.47 ± 2.25	5.23 ± 1.85	< 0.001
IOP post-goniopuncture			
Range	15–30	12–30	
mean ± SD	21.95 ± 4.04	18.68 ± 5.40	0.03
Changes of IOP, mean ± SD	−6.14 ± 6.59	−10.05 ± 4.02	0.03

*IOP* intraocular pressure, *SD* standard deviation

^*^Two-tailed student t test

Comparing the preoperative and 2 years postoperative results in each group, improvement regarding visual acuity, decrease in antiglaucoma drug use and decrease in IOP were all statistically significant (Table 3).

**Table 3:** Preoperative and 2 years postoperative results

Parameters	Phaco-viscocanalostomy group (*n* = 39)			Phaco-viscocanalostomy with Ologen® implant group (*n* = 40)		
	Preoperative	Postoperative	*P* value	Preoperative	Postoperative	*P* value
Visual acuity						
Range	0.1–0.7	0.5–1.0		0.1–0.6	0.5–1.0	
mean ± SD	0.41 ± 0.18	0.75 ± 0.18	< 0.001^†^	0.37 ± 0.17	0.73 ± 0.19	< 0.001^†^
IOP^a^; mmHg						
Range	15–25	15–22		14–26	13–21	
mean ± SD	19.46 ± 3.26	16.85 ± 1.58	< 0.001^†^	19.18 ± 3.19	15.96 ± 1.79	< 0.001^†^
Antiglaucoma drugs; n						
Range	2–4	0–4		2–4	0–4	
mean ± SD	2.85 ± 0.87	0.64 ± 1.01	< 0.001^‡^	2.88 ± 0.91	0.35 ± 0.86	< 0.001^‡^

*IOP* intraocular pressure, *SD* standard deviation

^a^measured with treatment

^†^One-tailed student t test

^‡^Man-Whitney U test

Two years postoperatively, the mean IOP showed a statistically significant decrease in the OloPhacovisco group compared to the Phacovisco group (*p* = 0.02), the use of antiglaucoma drugs was not significantly different between the two groups (*p* = 0.06), there was a statistically significant higher complete success rate in the OloPhacovisco group than the Phacovisco group (*p* = 0.04). Regarding eyes classified as failures, the results were not significantly different between the two groups (*p* = 0.67) and the results of visual acuity were not significantly different between the two groups (*p* = 0.63) (Table 4).

**Table 4:** Results 2 years postoperatively

Parameters	Phaco-viscocanalostomy group (*n* = 39)	Phaco-viscocanalostomy with Ologen® implant group (*n* = 40)	*p* value
Visual acuity			
Range	0.5–1.0	0.5–1.0	
mean ± SD	0.75 ± 0.18	0.73 ± 0.19	0.63^†^
IOP^a^; mmHg			
Range	15–22	13–21	
mean ± SD	16.85 ± 1.58	15.96 ± 1.79	0.02^†^
Antiglaucoma drugs; n			
Range	0–4	0–4	
mean ± SD	0.64 ± 1.01	0.35 ± 0.86	0.06^‡^
Success results			
complete success, n (%)	23 (59.0)	32 (80.0)	0.04^§^
qualified success, n (%)	12 (30.8)	5 (12.5)	0.048^§^
failure, n (%)	4 (10.3)	3 (7.5)	0.67^§^

*IOP* intraocular pressure, *SD* standard deviation

^a^measured without treatment

^†^Two-tailed student t test

^‡^Man-Whitney U test

^§^Chi square test

## Discussion

Non-penetrating glaucoma surgeries have a significant IOP reducing effect and offer some advantages over the conventional gold standard trabeculectomy for minimizing postoperative complications, such as excess filtration that may lead to prolonged flat anterior chamber, persistent hypotony and choroidal detachment and bleb-related complications, e.g. encysted bleb that may lead to late failure and bleb-related infections [1–4]. This study showed that there was a significant improvement of visual acuity, a significant reduction in IOP and a significant decrease in the use of antiglaucoma medications between the preoperative and postoperative results in both groups. At 2 years postoperatively the biodegradable Ologen® implant improved the success of phaco-viscocanalostomy, the IOP reduction was statistically significant in the OloPhacovisco group compared to the Phacovisco group. In addition, the number of cases with complete success were significantly higher in the OloPhacovisco group than the Phacovisico group. Since the Ologen® implant is porous, it allows aqueous fluid to percolate through it to reach the two ostia created in Schlemm’s canal. The porous structure of Ologen® allows fibroblasts and myofibroblasts to grow into the pores and secrete a loose connective tissue matrix to reduce scar formation and wound contraction, which leads to improvement of surgical results.

The main site of resistance to aqueous outflow has been demonstrated at the site of the juxtacanalicular trabecular meshwork and the inner wall of Schlemm’s canal [5]. In viscocanalostomy, the removal of a part of this tissue during surgery will help to decrease IOP, which also can be achieved by restoring the normal trabecular channels through the Schlemm’s canal by viscoelastic dilatation of the canal and creating two ostia of the canal at the site of the removal of the deep scleral flap through which aqueous fluid can flow circumferentially through the canal to the aqueous vein. Another mechanism that helps aqueous drainage, as described by Delarive et al. [6], occurs in the interscleral space. They showed that new aqueous humor drainage vessels grow in the interscleral space and absorb aqueous fluid flowing through the trabeculo-Descemet window. The presence of Ologen® implant in this bleb can extend its survival and prevent its collapse. Another route for aqueous humor outflow in is into the suprachoroidal space, which can be achieved by removal of 90% of the sclera during the deep scleral dissection [7].

In the original viscocanalostomy by Stegmann et al [8], they injected Healon GV into the subscleral lake after the tight closure of the superficial scleral flap to decrease fibrin cross-linking, and early scarring and failure. The biodegradable Ologen® implant may be a better alternative to Healon GV as it lasts 3–6 months before degradation. Many authors have used the biodegradable implants successfully as a spacer in deep sclerectomy to prevent collapse of the superficial flap and decrease postoperative fibrosis [9–13]. The IOP lowering effect of viscocanalostomy in long-term periods has been confirmed by many studies [14–16]. The IOP lowering effect of non-penetrating glaucoma surgeries has been found to comparable to, but not better than, trabeculectomy, but non-penetrating surgeries cause fewer surgical complications [2–9]. The IOP lowering effect of Ologen® implant as an adjuvant to trabeculectomy has been confirmed in many studies [1–3, 17].

Goniopuncture markedly improves the success rate of the surgical results. It makes a direct passage for aqueous fluid from the anterior chamber to the intrascleral lake. The Ologen® implant makes Nd:YAG laser goniopuncture much easier and more effective, and can be performed using a lower number of laser shots. This was attributed to the presence of the Ologen® implant stretching the Descemet’s membrane and making its opening with Nd:YAG is much easier as well as facilitating its viewing by enhancing the contrast behind the Descemet’s membrane.

Non-penetrating glaucoma surgeries give results comparable to trabeculectomy regarding IOP control, but with fewer complications [4, 9, 15, 16, 18–20]. It requires a longer learning curve, but it is safer than trabeculectomy. Dehan and Drusedau [21] recommended non-penetrating glaucoma surgeries as a first line of treatment before medical therapy, especially in young patients. To our knowledge, this is the first study to use Ologen® implant in viscocanalostomy, which may broaden the scope for further work in this field.

The main limitations of this study are the relatively small sample size and short follow-up period.

## Conclusions

The use of an adjunctive Ologen® implant with phaco-viscocanalostomy was found to be safe, and it provided more IOP reduction than phaco-viscocanalostomy alone. Although good success rate is achieved with visco-canalostomy especially with Olo group, goniopunture is very commonly required.

Larger randomized trials with longer follow-up periods are needed to confirm the safety and efficacy of this device in viscocanalostomy.

## Abbreviations

IOP: Intraocular pressure
Nd:YAG: Neodymium: yttrium-aluminum-garnet
OAG: Open angle glaucoma
OloPhacovisco group: Phaco-viscocanalostomy with Ologen® implant group
Phacovisco group: Phaco-viscocanalostomy group
POAG: Primary open-angle glaucoma
SPSS: Statistical package for social science
VA: Visual acuity
χ^2^: Chi square
